# Increased functional connectivity after stroke correlates with behavioral scores in non-human primate model

**DOI:** 10.1038/s41598-017-07175-y

**Published:** 2017-07-27

**Authors:** Carlos R. Hernandez-Castillo, Joseph Y. Nashed, Juan Fernandez-Ruiz, Justin Wang, Jason Gallivan, Douglas J. Cook

**Affiliations:** 1 CONACYT – Instituto de Neuroetologia, Universidad Veracruzana, Xalapa, Mexico; 2 Centre for Neuroscience studies, Queen’s University, Kingston, Canada; 3 Departamento de Fisiologia, Facultad de Medicina, Universidad Nacional Autonoma de Mexico, Mexico, Mexico; 4 Department of Surgery, Faculty of Health Sciences, Queen’s University, Kingston, Canada

## Abstract

Here we characterized the functional connectivity (FC) changes occurring after a controlled MCA stroke in a primate model. We hypothesize that if FC can inform about the neural changes after a stroke in the non-human primate (NHP) stroke model, then significant FC changes after the stroke would have to correlate with the remaining behavioral capacities. Eleven cynomolgus monkeys underwent an experimental middle cerebral artery occlusion while five monkeys remained as the control group. One month later the neurological function was assessed with a set of fine motor tasks and the Nonhuman Primate Stroke Scale (NHPSS). Structural and functional connectivity analyses were done to compare both groups. Three FC changes showed significant behavioral correlations: right sensorimotor-right lateral intraparietal FC with the six-well task; left posterior intraparietal-left dorsal premotor FC with the hill task; and right visual-left primary motor FC with the NHPSS. In the three instances, stronger FC correlated with better behavioral outcome. The results show that the functional changes correlating with behavioral outcomes involved sensorimotor cortices that were not restricted to the affected hemisphere. These results show that the FC analysis in NHP stroke model is a relevant methodology suitable to inform the neural changes occurring after a stroke.

## Introduction

Animal models in biomedical research have had a paramount impact in treating a number of conditions. Specifically, non-human primate (NHP) models provide the capacity to test variables closely related to the human central nervous system function^1^. In this regard, a recent study successfully developed a gyrencephalic NHP stroke model using a middle cerebral artery (MCA) occlusion method, becoming the model that most closely resembles the most common stroke type in humans^2, 3^. This NHP model is ideal to study the brain response to a stroke using magnetic resonance imaging (MRI) (both structural and functional), as well as its relation to the remaining behavioral capacities of the individual.

Clinical manifestations of ischemia depend on the vascular territory deprived of blood flow, neurological localization of the tissue affected and the availability of collateral flow from adjacent vascular supply. Deficits can include cognitive impairment, sensory loss, weakness, and diminished motor control. The specific relationship between lesion location/size and behavioral outcome is complex because of the variability of the functional localization within the brain and because the adaptive response of the surviving tissue influences the rate and magnitude of the possible recovery^4^.

A number of approaches using imaging data have analyzed how the remaining brain functionality reorganizes following the loss of tissue after a stroke^5^. Initial functional task-related imaging studies found significant negative correlations between task-related brain activations and recovery across time^6^. However, recent studies have suggested more complex patterns of functional reorganization^7, 8^. This complexity in the functional reorganization after a stroke has also been observed via the analysis of resting state networks (RSN)^8, 9^. The analyses of RSN could help explain, for example, effects on distant areas not directly connected to the stroke via white matter, but belonging to the same functionally intrinsic network^10^. RSN analyses are not bound to performing a task within the MRI scanner, thus enabling them for direct comparisons between clinical findings and animal stroke models, especially with non-human primates (NHP)^11^.

Hence, the aim of the current study was to characterize the functional connectivity changes occurring after a controlled MCA stroke in the NHP model. This analysis was followed by an evaluation of the relationship between the FC changes with the remaining behavioral capacities of the NHP.

## Results

### Behavioral results

The experimental group had an initial Nonhuman Primate Stroke Scale (NHPSS) score of 0, that changed to a score of M = 9.9, SD = 8.23, one month after surgery. A paired-samples t-test was conducted to compare the NHPSS score before and after the stroke. The analysis showed that there was a significant difference between the scores (t(10) = 3.9, p = 0.002). A one sample t-test on the change in hand use in the 2-tube test (M = 0.4, SD = 0.27) also showed significant differences (t(10) = 5, p = 0.0005). A one-sample t-test on 6-well task performance (M = 15.7, SD = 6.79) revealed significant deficits (t (10) = 9.1, p < 0.00005). Finally, a paired-samples t-test showed that there were also significant differences in the hand use after the stroke as measured by the Hill task. The non-affected right hand had shorter completions times (M = 8.8, SD = 5.7) than the affected left hand (M = 32.9, SD = 11.6) (t(10) = 5.6, p = 0.0002).

### Structural imaging results

**Figure 1:**
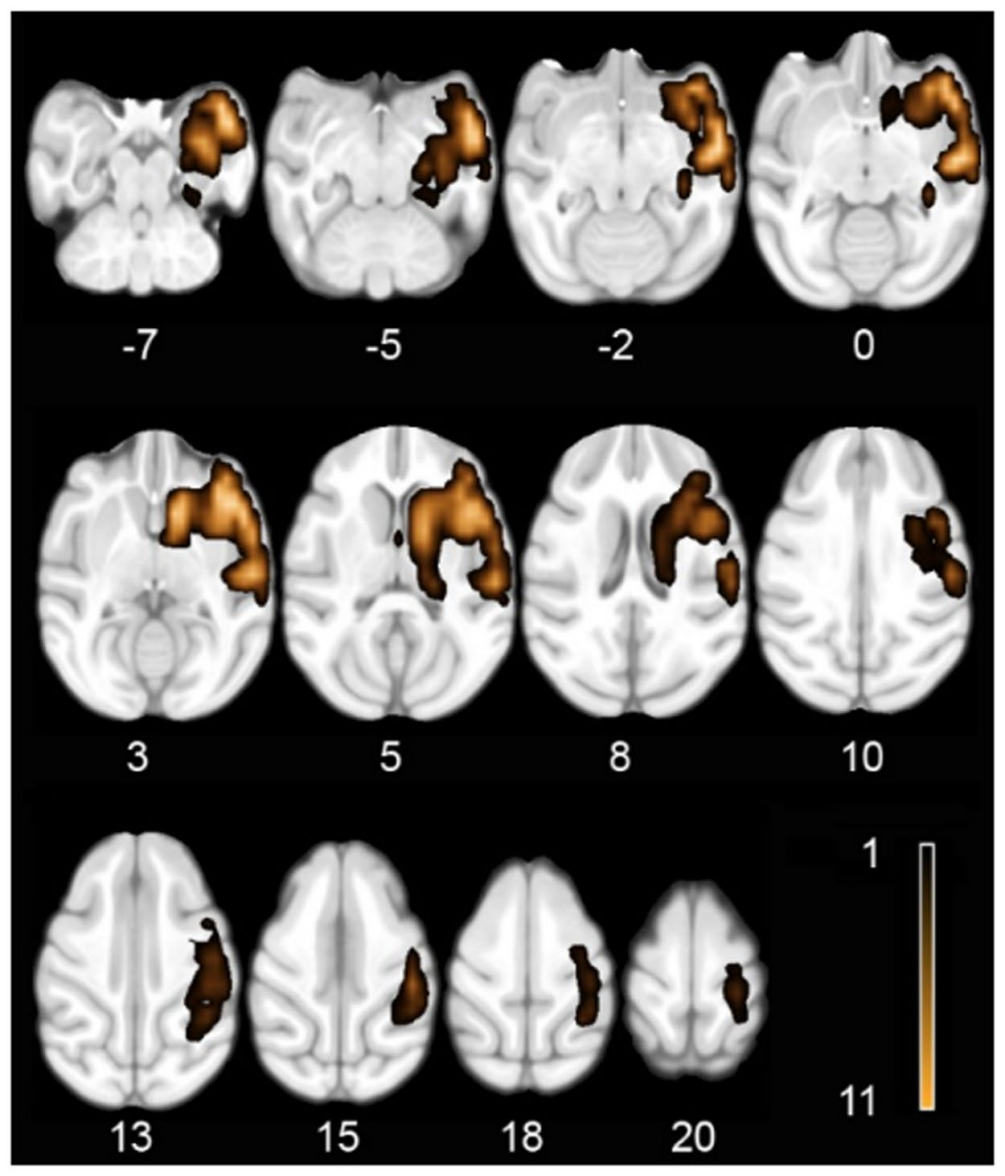
Horizontal sections including the z coordinate showing the stroke area mounted on the Montreal Neurological Institute (MNI) cynomolgus macaque atlas template. Note that the color intensity indicates the number of monkeys affected in the corresponding area as specified in the color bar.

Analyses of the lesions (Fig. 1) showed that they were consistent with the original NHP stroke model report^2^. The infarction volumes had a range between 14852 and 20814 mm3 with a mean of 18630 mm3 across the NHP stroke group. The regions most consistently affected across the eleven monkeys included the right hemisphere temporal pole, insular cortex, auditory cortex, somatosensory cortex, claustrum, ventral premotor cortex, caudate and putamen. It also should be noted that all NHP stroke group animals had damage to the anterior limb of the internal capsule (Fig. 1).

### Functional connectivity results

**Figure 2:**
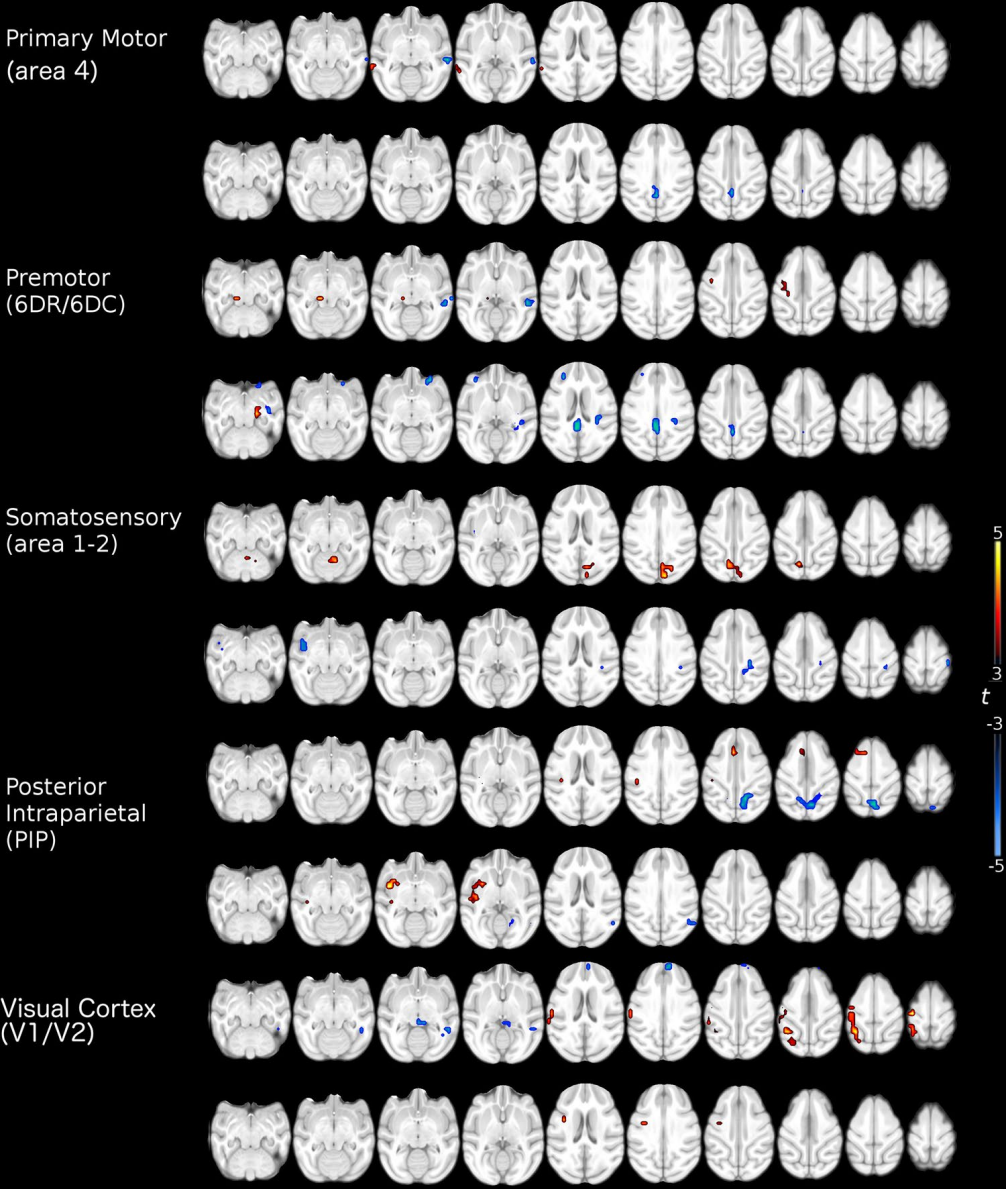
Significant functional connectivity differences between healthy controls and stroke primates. Warm colors indicate an increase and cold colors indicate a decrease in the functional connectivity on the stroke group (11 subjects). Maps corrected for multiple comparison using False Discovery Rate (FDR) p < 0.05.

**Figure 3:**
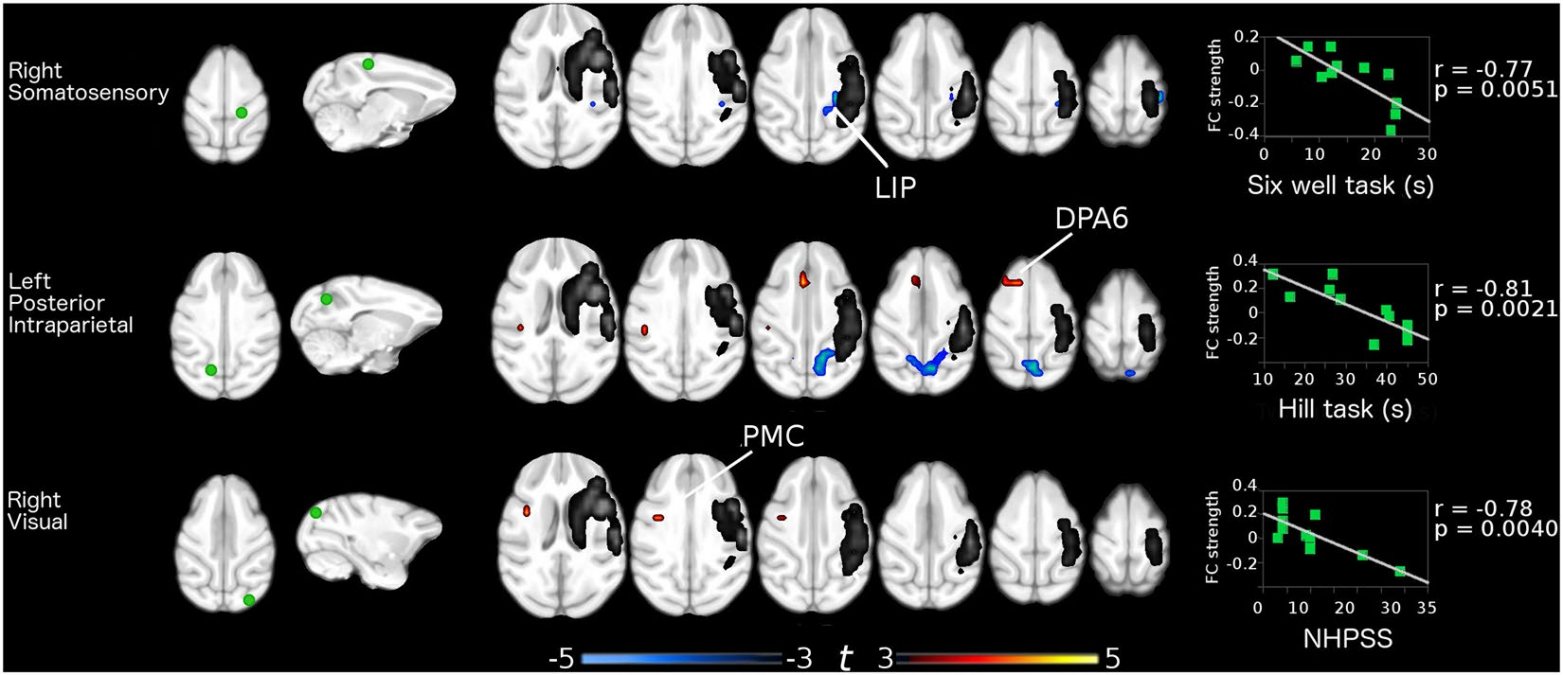
Functional connections showing significant correlation with behavioral scores. Left column shows the seeds location. Middle column shows the areas that had significant Functional Connectivity (FC) differences between groups that also correlated with the behavioral impairment as shown in the right column. The stroke area is showed in gray scale.

Based in an Independent component analysis (ICA) we select 10 regions to serve as seeds for the following analysis (Table 1. See methods). The FC analysis of the stroke group showed that each of the analyzed intrinsic networks presented significant differences with the control group (Fig. 2). Three functional connections correlated with the behavioral performance of the experimental group. The first was a decreased FC between the somatosensory area 1–2 and the lateral intraparietal area (LIP) ipsilateral to the lesion. This FC had a significant negative correlation with the performing times in the six well task (r = −0.79, p = 0.003) (Fig. 3, first row). The second was an increase in FC between the posterior intraparietal area (PIP) and the dorsal premotor area 6 (DPA6), both on the contralateral hemisphere of the stroke. This FC showed a significant negative correlation with the hill task performance using the left hand (r = −0.81, p = 0.002) (Fig. 3, second row). Finally, the third was also an increase in FC between the right visual and the left primary motor cortex (PMC). This FC had a significant negative correlation with the NHPSS scale (r = −0.78, p = 0.004) (Fig. 3, third row).

**Table 1:** Anatomical location of seed for functional connectivity analysis.

Side	Anatomical region	X	Y	Z
Left	Primary Motor Cortex (F1/Area 4)	−12	−4	20
Right	Primary Motor Cortex (F1/Area 4)	12	−4	20
Left	Premotor Cortex (6DR/6DC)	−6	0	20
Right	Premotor Cortex (6DR/6DC)	6	0	20
Left	Somatosensory (Area 1-2)	−6	−14	18
Right	Somatosensory (Area 1-2)	6	−14	18
Left	Posterior Intraparietal Area (PIP)	−8	−26	10
Right	Posterior Intraparietal Area (PIP)	7	−26	10
Left	Visual Cortex (V1/V2)	−10	−32	16
Right	Visual Cortex (V1/V2)	10	−32	16

Coordinates in MNI space from cynomulgus macaque atlas template in millimeters.

## Discussion

Our results show a large degree of stroke homogeneity across animals that resulted in a number of significant FC group changes. More importantly, our analyses uncovered FC changes that showed significant correlations with the behavioral outcome in the stroke group. Although these correlations varied in strength, connectivity laterality, or hemispheric localization, they all had in common that higher FC correlated with better behavioral performance. Following we provide a detailed discussion of our results.

A critical goal of this research was to test if FC changes correlated with the behavioral outcome in a chronic stage in the stroke NHP model previously designed by our group^2^. The results show three functional connections that correlated with the behavioral outcome. The first one was the connectivity between the right somatosensory areas 1–2 and right LIP, which showed a decrease in connectivity in relation to the control group. This reduction had a negative correlation with the performing time in the six-well task. That is, those subjects that maintained FC levels at the normal level, performed better than those that show connectivity reductions. The second functional connection was between the left PIP and the left DPA6, which showed a significant increase in relation to the control group. This connection had a negative correlation with the performance of the hill task with the left hand. Namely, those subjects that showed significant FC increases finished the task faster than those subjects that did not increase, or even decrease this functional connection. Finally, the functional connection between the right V1 and the PMC also was stronger in the stroke group compared to the control group. Again, those animals that had increases in their FC performed better in the NHPSS than those that did not change.

In these three instances where FC changes correlated with a behavioral measure, those animals that had higher FCs performed better than those that either did not change or decreased their FC. Although much research is needed to understand the nature of these changes, it is possible to suggest a preliminary interpretation. In the case of the first change between somatosensory areas 1–2 and LIP, the net FC change was a decrease. It can be speculated that those animals that preserved the normal FC levels performed better than the rest of the group. Similar findings have been reported in an aphasic stroke study where it was shown that aphasic patients with preserved intertemporal connectivity displayed better receptive language function^12^. In contrast, the other two connectivity changes were above the control group, suggesting that those animals that showed increased connectivity levels were able to compensate the stroke effects, while those that did not show the increase performed worse. Compensatory changes have been found in different conditions including neurological^13–15^ or psychiatric^16^ disorders. Stroke studies have also found evidence suggesting FC compensatory changes, e.g. it has been suggested that increases in interhemispheric M1 FC may play a beneficial role during motor recovery^17^. The interpretation of these last changes as a compensatory mechanism, however, needs to be taken with caution since this result is correlative.

The areas involved in the FC changes that showed correlations with behavioral measures are fundamental for visuomotor behavior. This is important because the tested behaviors critically rely on visuomotor function. V1, LIP and PIP, are involved in visual processing and visuomotor transformations critical in the spatial representation of the external world^18, 19^; somatosensory areas 1–2, are fundamental for tactile perception and object manipulation^20^; DPA6, is involved in precision grasping^21^; and PMC, which is the main output of the cortical motor system^22^.

The intrinsic FC analysis revealed a large number of combinations of laterality and hemispheric localization connectivity (Fig. 2), including the three connections analyzed here. First, the somatosensory area 1–2 seed and the target LIP area were both on the right hemisphere, ipsilateral to the stroke. This hemisphere has the significant burden of the stroke damage, although after one month these processes should have had stabilized^2^. This functional connection decreased in relation to the control group, and those animals that maintained normal FC levels performed better than those that showed decreases. Similar results in patients have shown FC connectivity reductions in the somatosensory network ipsilateral to the stroke associated with sensory deficits^23^. The second connection was seeded in the left PIP and the target area was on the left DPA6. Therefore, this putatively compensatory FC increase took place completely in the non-stroked hemisphere, highlighting at the same time, the value of the NHPSS to assess not only the damage on the hemisphere with the stroke, but the correlation with the changes occurring in the spared one. This finding is supported by previous reports showing changes in the non-lesioned hemisphere associated to functional recovery not only in stroke, but also in another lesion models^24–26^.

The final FC change was again a possible compensatory increase in relation to the control group, but this was seeded in V1 of the stroked right hemisphere, and the target was the PMC of the left hemisphere. This was the only interhemispheric FC change that correlated with the behavioral outcome. An influential report analyzing resting state networks found that disruptions of interhemispheric FC in the attention and somatomotor networks were correlated with motor impairments, while FC intrahemispheric deteriorations were not^27^. Our results support the value of interhemispheric FC, however, they also support a possible role for intrahemispheric connections in both healthy and stroked hemispheres.

A limitation of our study is that this is an animal model where we can only measure a small repertoire of behaviors. With lesions as large as the ones produced by MCA strokes, other functions not tested were probably also affected. This could have an impact on the limited number of correlations found between FC changes and behavioral performance. There are also limitations related to the imaging technique, including the possible effect of the use of anesthesia on brain connectivity, although previous findings suggest that the functional connectivity topographical structure remains similar across different depths of anesthesia^28^. Another limitation relates to the method used to analyze the functional connectivity changes. Here we decided to use ICA to localize ROIs related to each functional network, however, different approaches can also be applied to find both extended or more focalized changes. This can be done taking advantage of different methodologies such as increasing the number and/or the area of ROIs as well as using functional/anatomical parcellations^29^.

## Conclusion

Our results show significant FC changes after a MCA stroke. We identified putatively functional compensatory FCs involving contralateral and interhemispheric connections. We also identified an ipsilateral FC decrease that correlates to poor recovery. These results show that the analysis of resting state networks in a NHP stroke model is a relevant methodology within the stroke translational medicine field that could be implemented as a biomarker for novel pharmaceutical therapies, or to inform the possible implementation of brain-computer interfaces to restore functions after stroke.

## Materials and Methods

### Subjects

Sixteen healthy adult cynomolgus monkeys (Macaca fascicularis; male captive-bred; 2.4–5.0 kg) were tested in this study. Eleven monkeys underwent an experimental model of surgical middle cerebral artery occlusion (MCAO)^2^; five monkeys remained as the control group. All the surgical and experimental procedures were carried out in accordance with the Canadian Council of Animal Care policy on the use of laboratory animals and approved by the Animal Use Subcommittee of Queen’s University Council on Animal Care.

### Stroke model

Animals were anesthetized (Isoflurane 1.0–2.5%), intubated and ventilated. Non-invasive monitoring included BP by leg cuff, end-tidal CO2, O2 saturation, ECG and temperature by rectal probe. Temperature was maintained (37 ± 0.5 °C) by heating blanket. A femoral arterial line was used to monitor BP and blood gases. MCAO in cynomolgus macaques was performed using a right pterional craniotomy and occluding the right MCA in the Sylvian fissure with a 5mm titanium aneurysm clip proximal to the orbitofrontal branch. At the end of the MCAO, the aneurysm clip was removed in order to restore blood flow as described previously^30^.

### Neurological assessments

The neurological function was assessed one month after the MCAO procedure using the hill and valley task, the two‐tube task, the six-well task^31, 32^, and the Nonhuman Primate Stroke Scale (NHPSS). The NHPSS score is a composite rate of state of consciousness, defense reaction, grasp reflex, extremity movement, gait, circling, bradykinesia, balance, neglect, visual field cut/hemianopsia and facial weakness, many of which are also incorporated in the NIH Stroke Scoring system in humans^33^. From a total of 41 points, 0 corresponds to normal behavior and 41 to severe bilateral neurological impairment. Previous experiments in 5 macaques subjected to a 90 min MCAO showed an initial peak in NHPSS scores that persisted for the first 36 hours and then gradually dropped to a plateau between 14 and 30 days, suggesting that the behavioral impairment had become stable at one-month post stroke^2^.

### MRI acquisition

Magnetic resonance scanning was performed at the Queen’s University Centre for Neuroscience Studies using a 3 T Siemens Trio scanner. For the acquisition of MRI images, the animals were anesthetized (Isoflurane 1.0–2.5%), intubated and ventilated. Non‐invasive monitoring included BP by leg cuff, end‐tidal CO2, O2 saturation, ECG and temperature by rectal probe. Temperature was maintained (37 ± 0.5 C) by heating blanket. A femoral arterial line was used to monitor BP and blood gases. It should be noted that several studies have tested the reliability of BOLD signal in animals under anesthesia, comparing different anesthetics including isoflurane during task and task free (resting state) conditions^34^. Furthermore, studies in NHP have shown the correspondence between the resting state networks of NHP and humans under the effects of anesthesia^35^.

The acquisition consisted of a high-resolution, T1-weighted, magnetization-prepared rapid gradient echo (MP-RAGE TR  =  1600 ms; TE  =  3.92 ms, flip angle  =  9°, matrix = 320 × 320, FOV 192 × 192, 120 slices and final voxel size = 0.6 × 0.6 × 0.6 mm. Resting state fMRI data were acquired using a gradient-echo echo-planar sequence sensitive to BOLD contrast, volume repetition time (TR)  =  2000 ms, T2* echo time (TE)  =  28 ms, flip angle  =  80°, slices matrix = 64 × 64, FOV = 768 × 768, 28 slices and a final voxel size = 2 × 2 × 2 mm. In all monkeys, the slices were acquired using contiguous, interleaved acquisition with two runs of 353 functional volumes each (approximately 17 min).

### Independent component analysis

In order to localize regions of interest to be used as anchor seed in subsequent analysis, independent component analysis (ICA) was carried out in the control group using FSL’s MELODIC^35^. Preprocessing consisted of motion correction, removal of non-brain tissue, spatial smoothing using a 3 mm full-width-at-half-maximum Gaussian kernel, and high-pass temporal filtering equivalent to 100 seconds (0.01 Hz). After preprocessing, the fMRI volumes were registered to the subject’s high-resolution T1-weighted scan using a rigid body registration and then to Montreal Neurological Institute cynomolgus macaque atlas template^36^ using affine registration with a warp resolution of 2mm. The data set was decomposed into 30 independent components^37^. These components comprised several intrinsic functional networks and image artifacts like movement, physiological noise, and cerebrospinal fluid flow. Five components of interest related to visuomotor processing were selected by visual inspection based on previous literature^38^ and by the frequency spectra of the time courses of the components. These components included the primary motor, premotor, somatosensory, intraparietal and primary visual. Local maxima of each component selected were used as seeds for the functional connectivity analysis (Table 1).

### Lesion analysis

To assess the stroke area and exclude it from subsequent functional connectivity analyses we created a composite image for the stroke group. After spatial normalization to the cynomolgus brain template, each monkey T1 image was segmented into gray matter (GM), white matter (WM) and cerebrospinal fluid (CSF) using FSL. The CSF segmentation of the stroke group was binarized and then added together to create an image of the region affected by the stroke where the intensity represents the occurrence of the lesion in the group (Fig. 1). The CSF segmentation of the control group was then used to remove the regular regions with CSF.

### Resting-state preprocessing

rsfMRI preprocessing included brain extraction, time shifting, motion correction, spatial smoothing (3 mm full-width at half-maximum Gaussian kernel), linear trend removal, and temporal filtering (band pass, 0.0025–0.05 Hz) using FSL (FMRIB, Oxford University, Oxford, UK). A regression technique was used to remove sources of variance including white matter, cerebrospinal fluid, and movement rate after motion correction^39^. Using FSL’s FNIRT, all structural T1-weighted images were warped to the Montreal Neurological Institute cynomolgus macaque atlas template^36^. After rigid alignment of rsfMRI images to the corresponding structural images, spatial normalization of rsfMRI images to the MNI template was achieved using the transformation field acquired during the structural image registration. To remove any signal from the stroke area, all functional images were masked using the stroke mask previously generated.

### Functional Connectivity analysis

Based on the ICA results, we identified the local maxima of intrinsic functional networks in the control group to use them as anchor seeds for the next procedure in the experimental group (Table 1). The mean time course of each defined seed was extracted by calculating the average of all voxels within a 6-mm sphere (MATLAB R2014b, The Mathworks, Inc., Natick, MA). Functional connectivity maps were created by calculating a Pearson’s linear correlation between the seed’s average signal and every other voxel in the brain excluding those labeled as stroke-lesion. Mean functional connectivity maps were created for both groups and overlapped for visual comparison. A two-sample t-test was performed between the two groups’ functional connectivity maps to detect significant differences. Functional connectivity maps were corrected for multiple comparisons at the whole brain level setting a significance p value of p < 0.05 corrected using false discovery rate (FDR)^40^. For the experimental group, the average functional connectivity strength of each significant cluster was calculated. Then, Pearson’s correlations between those functional abnormalities and the behavioral tests scores were obtained. Finally, the correlation significance was set at the p value < 0.05 FDR corrected.
